# Erythropoietin Attenuates the Brain Edema Response after Experimental Traumatic Brain Injury

**DOI:** 10.1089/neu.2017.5015

**Published:** 2018-02-15

**Authors:** Jonas Blixt, Eli Gunnarson, Michael Wanecek

**Affiliations:** ^1^ Perioperative Medicine and Intensive Care, Karolinska University Hospital, Karolinska Institutet, Stockholm, Sweden.; ^2^ Department of Physiology and Pharmacology, Karolinska University Hospital, Karolinska Institutet, Stockholm, Sweden.; ^3^ Department of Women's and Children's Health Karolinska University Hospital, Karolinska Institutet, Stockholm, Sweden.

**Keywords:** aquaporin, BBB, brain edema, EPO, TBI

## Abstract

Erythropoietin (EPO) has neuroprotective effects in multiple central nervous system (CNS) injury models; however EPO's effects on traumatic brain edema are elusive. To explore EPO as an intervention in traumatic brain edema, male Sprague–Dawley (SD) rats were subjected to blunt, controlled traumatic brain injury (TBI). Animals were randomized to EPO 5000 IU/kg or saline (control group) intraperitoneally within 30 min after trauma and once daily for 4 consecutive days. Brain MRI, immunohistofluorescence, immunohistochemistry, and quantitative protein analysis were performed at days 1 and 4 post- trauma. EPO significantly prevented the loss of the tight junction protein zona occludens 1 (ZO-1) observed in control animals after trauma. The decrease of ZO-1 in the control group was associated with an immunoglobulin (Ig)G increase in the perilesional parenchyma, indicating blood–brain barrier (BBB) dysfunction and increased permeability. EPO treatment attenuated decrease in apparent diffusion coefficient (ADC) after trauma, suggesting a reduction of cytotoxic edema, and reduced the IgG leakage, indicating that EPO contributed to preserve BBB integrity and attenuated vasogenic edema. Animals treated with EPO demonstrated conserved levels of aquaporin 4 (AQP4) protein expression in the perilesional area, whereas control animals showed a reduction of AQP4. We show that post TBI administration of EPO decreases early cytotoxic brain edema and preserves structural and functional properties of the BBB, leading to attenuation of the vasogenic edema response. The data support that the mechanisms involve preservation of the tight junction protein ZO-1 and the water channel AQP4, and indicate that treatment with EPO may have beneficial effects on the brain edema response following TBI.

## Introduction

Traumatic brain injury (TBI) is a major cause of early death and neurological disability throughout the world and remains a major public health problem.^1,2^ Injury mechanisms in brain trauma include several cascades of events such as release of excitotoxic molecules, metabolic challenges, inflammation, and apoptosis, accompanied by breakdown of the blood–brain barrier (BBB) and cell swelling, which can finally lead to cell death. Despite several recent clinical studies based on promising experimental evidenc targeting injury mechanisms, there are currently no available pharmacological treatments that convincingly counteract secondary injuries and/or improve long-term functional recovery after human TBI.^3–9^

Erythropoietin (EPO) is a glycoprotein cytokine that has been investigated in TBI research. EPO is produced mainly in the kidney, but also locally in brain cells under hypoxic conditions.^10,11^ EPO receptors have been found throughout the brain in various cell types such as neurons, astrocytes, and endothelial cells.^12–14^ In the literature, there is a substantial body of evidence in support of non-hematopoietic, neuroprotective effects of EPO^11,15^ in models of central nervous system (CNS) injury such as ischemia, trauma, intracranial hemorrhages, and diffuse axonal injuries. The proposed mechanisms include decreased inflammatory response,^16–18^ decrease of oxidative stress and anti-apoptotic effects.^16,19,20^

The effects of EPO treatment in clinical CNS lesions/injuries have varied. A recent randomized, prospective, placebo-controlled clinical trial in ischemic stroke showed that EPO significantly improved long-term neurological outcome.^21^ Following moderate or severe TBI, results on EPO treatment have been conflicting. One study found decreased mortality and improved neurological outcome,^22^ whereas the recent study by Nichol and colleagues did not find that EPO resulted in significant improvement of functional outcome when assessed 6 months after TBI.^23^

The clinical course that follows severe TBI is characterized by brain edema development and an associated increase in intracranial pressure contributing to most of the early deaths following trauma. Using a TBI model we have previously shown that the dynamic course of edema development include co-existing vasogenic and cytotoxic brain edema.^24^ The aim of the present study was to investigate whether EPO treatment can modulate the dynamic brain edema response following experimental focal brain injury.

## Methods

### Animals

Animal care and experimental procedures were conducted in accordance with the European Communities Council Directive of November 24, 1986 (86/609/EEC). Experimental protocols were approved by the Northern Stockholm Laboratory Animal Review Board. All studies were performed on male Sprague–Dawley rats (Scanbur, Sweden) 250–280 g, housed at the Karolinska University Hospital (Stockholm, Sweden) in polystyrene cages containing aspen wood shavings, and with free access to water and standard rodent chow and regulated 12 h light–dark cycles. All procedures and housing throughout the study were at room temperature (21°C ± 1°C) with normal air humidity.

### Experimental groups

A total of 75 male rats were used in this study and were randomized by drawing numbers. They were divided into a naïve group with animals without TBI, a control group that received NaCl after trauma, and a treatment group that received EPO after trauma. The naive group (*n* = 15) was further divided in groups of five to be used in different experiments. The NaCl or EPO groups (*n* = 30/group), were further randomized to be euthanized after 1 day or 4 days (*n* = 5/group/day) in three different experimental settings.

### Drug and dose

EPO (recombinant human Erythropoietin, Eprex, 4000 IU/ml, Janssen-Cilag, France) was administered intraperitoneally 30 min after trauma, and thereafter once daily in a dose of 5000 IU/kg throughout the study. Animals in the control group received an identical volume of saline (isotonic NaCl 0.9%, Fresenius Kabi, Sweden) intraperitoneally.

### TBI model

After randomization to either the naïve or the TBI group, the TBI rats were anesthetized by intramuscular injections of 0.15 mL of Hypnorm^®^ (10 mg/mL fluanisone and 0.315 mg/mL fentanyl citrate; Janssen Pharmaceutical, Beerse, Belgium) and 0.2 mL Dormicum^®^ (midazolam, 1 mg/mL, F. Hoffmann-La Roche, Basel, Switzerland). Before skin incision, 0.1 mL of Marcain-Adrenalin^®^ (bupivacaine 5 mg/mL and adrenaline 5 μg/mL, Astra Zeneca, Södertälje, Sweden) was injected subcutaneously in the sagittal midline of the skull. The rats were placed in a stereotactic frame. Eye-gel (Fucithalmic®) applied for eye protection and isotonic saline (NaCl 9mg/mL) were used to clean and rinse the scalp wound throughout the experiment. Under aseptic conditions, a craniotomy (2 mm in diameter) was drilled under microscopic guidance at a point 2–3 mm posterior and 2–2.5 mm lateral to the right side of bregma. A standardized parietal contusion was produced by a weight-drop model after Feeny that has been further developed in our laboratory.^25,26^ A weight of 24 g was dropped from a height of 7 cm on a 1.8 mm diameter piston that was set to allow for a maximal tissue compression of 3 mm. The craniotomy was not resealed. Spontaneous ventilation was monitored by pulse oximetry (Datex-Ohmeda, Helsinki, Finland), and oxygen was supplied through a facemask to keep oxygen saturation above 90%. The body temperature was kept at 37.5–38°C by means of a feedback-regulated heating pad. Heart rate and respiratory rate were continuously observed and within physiological range throughout the experimental period (Table 1). The skin was sutured using Vicryl 4-0 (Ethicon, Johnson & Johnson, New Brunswick, NJ) and the animals were allowed to recover under at heating lamp with an extra supply of oxygen. The TBI animals showed no deficit in motor, balance, or sensory function during the post-traumatic phase, and they showed normal grooming and feeding behavior.

**Table 1.** Physiological Data during Operation

*Group*	*Naive*	*Control*	*EPO*
Heart rate	351 ± 11	359 ± 10	360 ± 7
Respiratory rate	80 ± 2	85 ± 3	83 ± 5
Saturation	94 ± 3	95 ± 1	94 ± 2
Temperature (°C)	37.5–38	37.5–38	37.5–38
Body weight (g)	258 ± 5	252 ± 4	255 ± 3

EPO, erythropoietin.

### Euthanasia

Animals intended for immunohistochemistry were anaesthetized and euthanized by transcardial perfusion. All other animals were anaesthetized as described, and then immediately killed by decapitation.

### MRI

MRI of the brain (Naïve *n* = 5, Control/EPO 1 and 4 days *n* = 5/day/group) was performed at 1 day and 4 days post-injury. Animals were re-anaesthetized with the same doses of midazolam and Hypnorm. After intubation^27^ with a16 gauge intravenous catheter (45 mm length, 20 mm hub, 1.7 mm internal diameter [ID]), anaesthesia was maintained with 1.5–2% isoflurane, and animals were mechanically ventilated with a small animal ventilator SAR-830P (Stoelting Co, Chicago, IL). The ventilator was set at a tidal volume of 8 mL/kg and a respiratory rate of 40 breaths/min. Rectal temperature was maintained between 36.5°C and 37.5°C by using a feedback-regulated heating pad. The eyes were lubricated and covered to prevent dehydration. MRI was performed with a Bruker Biospec 4.7 T scanner (Bruker, Karlsruhe, Germany) fitted with a 12 cm ID self-shielded gradient system (max. gradient strength 200 mT/m). A 38 mm diameter head coil birdcage was used for imaging. Qualitative T2-weighted images were obtained using a rapid acquisition with relaxation enhancement (RARE)^28^ (repetition time [TR] = 2500 ms; effective echo time (TE) = 30 ms; RARE factor = 8; field of view (FOV) = 3.5 × 3.5 cm; matrix 256 × 256) five contiguous slices of 1 mm, two averages.

At the center of the lesion, a 1.5 mm thick diffusion-weighted image was taken. In order to reach an acceptable signal-to-noise ratio without dramatically increasing the acquisition time, a 128 × 64 matrix for FOV = 3.5 × 3.5 cm was used. Diffusion images were obtained using a pulsed gradient stimulated echo sequence with six diffusion weighting factor bs = 39.049, 93.557, 274.708, 401.350, 665.853, 2391.773 sec/mm^2^. The diffusion-sensitizing gradient was placed only along the direction of the slice selection gradient (i.e., rostrocaudally).

### MRI: Midline shifts (MLS)

MLS were determined on T2-weighted images with the use of analysis software ImageJ® 1.48v (Rasband, W.S., ImageJ, U. S. National Institutes of Health, Bethesda, MD). After optical adjustment of contrast and brightness, the distance between the outer border of the cortex and the middle of the third ventricle was measured from the ipsilateral (I) and contralateral (C) sides. MLS was calculated using the equation MLS = (I-C)/2.

### MRI: Edema analysis

Apparent diffusion coefficient (ADC) maps were calculated by ImageJ and the use of MRI Analysis Calculator, a plugin provided for free by Karl Schmidt. Further analysis was performed in the region of interest (ROI), with ROIs being placed in the perilesional cortex area, and at the same site in the contralateral hemisphere. Assessments of both MLS and ADC were performed blinded by one investigator, and calculated as mean of three repeated measurements. Repeated analysis with >2 weeks' separation did not show any statistical significant difference.

### Immunohistochemistry: Immunoglobulin (Ig)G staining protocol

Transcardial perfusion was performed (Naïve *n* = 5, Control/EPO 1 and 4 days *n* = 5/day/group) with cooled saline followed by cold 4% paraformaldehyde (PFA) in 0.15 M phosphate buffer (pH 7.3–7.4). Brains were removed and post-fixed for 90 min in the same fixative followed by wash overnight in 0.15 M phosphate buffer with 17% (w/v) sucrose at 4°C, thereafter snap frozen in isopentane and stored at −70°C. Whole brain sections 14 μm were cut horizontally through the length of the contusion using a Leica cryostat, and then thaw mounted onto Super Frost/Plus object glasses, dried at room temperature for 1–2 h and stored at −20°C prior to use.

The sections were incubated in 0.3% hydrogen peroxide for 30 min to quench endogenous peroxidase. Before incubation with a primary antibody, blocking serum was used to prevent nonspecific conjugate binding. An avidin-biotin blocking step was then performed with Vectastain Elite avidin-biotinylated enzyme complex (ABC) peroxidase kit (Vector Laboratories, Burlingame, CA) to prevent nonspecific conjugate binding to endogenous biotinylated proteins. Sections were then incubated with primary anti-IgG antibody overnight at +4°C. In negative controls (data not shown) slides were incubated with phosphate buffered saline (PBS). After washing, the indirect peroxidase method was used for detection of the primary antibodies. Biotin-conjugated donkey anti-goat immunoglobulin was used as conjugate. Sections were incubated with a standard ABC kit, and the bound peroxidase was detected via incubation with a 3'-diaminobenzidine (DAB) substrate kit. Sections were then dehydrated and mounted with DPX (Distrene 80, di-butyl phthalate, xylene, BDH Laboratory Supplies Pool, UK).

### Immunohistochemistry: IgG analysis

Stained sections were scanned with a Nikon Super Coolscan 4000 ED (32 bit, 4000 dpi, standard red, green blue [sRGB]) and saved as TIF images. After adjustment of contrast and brightness in ImageJ, parameters then remained unchanged through the series, and the pictures were converted to grayscale. Three ROIs of the same size were placed in representative areas in both hemispheres in three consecutive sections per animal, and the mean staining intensity for each animal was calculated. We chose to measure the monochromatic intensity of the IgG signal rather than the area, as the dissemination of IgG could be affected unevenly by local factors; for example, edema. We analyzed the contralateral side in all sections, both within groups, between groups, and against sections from naïve animals without any statistical differences in staining intensity. To diminish potential effects of small differences in staining, corresponding areas in the contralateral hemisphere were used for signal normalization, and the results were presented as ipsilateral/contralateral values (“ipsi/contra”).

### Immunohistofluorescence: Staining protocol

The brains for immunohistofluorescence analysis (Naive *n* = 5, Control/EPO 1 and 4 days *n* = 5/day/group) were quickly removed, snap frozen in isopentane, and stored at −70°C. Whole brain 14 μm cryosections were cut coronally through the center of the impact using a Leica cryostat (CM 3000, Leica Instruments GmbH, Nussloch, Germany). The sections were thaw mounted onto Super Frost/Plus object glasses (Menzel–Gläzer, Braunschweig, Germany) dried at room temperature for 1–2 h, and stored at −20°C prior to analysis.

Prior to staining, the sections were dried at room temperature for 30 min, rehydrated in PBS, and fixed in 4% buffered paraformaldehyde at room temperature for 10 min and then rinsed in PBS. Treatment for 60 min with blocking serum (PBS, 1% bovine serum albumin, 0.3% triton X-100) was used to prevent nonspecific conjugate binding to the primary antibodies. Primary antibodies (anti- zona occludens 1 [ZO-1] or anti-aquaporin [AQP]4) were diluted in blocking serum and added for incubation overnight at +4°C either in solitude or in mixtures. Sections were washed in PBS, incubated for 1 h with secondary antibodies, washed again, and cover-slipped with ProLong Gold anti-fade reagent with DAPI (Invitrogen Life Technologies, Carlsbad, CA, USA). Negative control staining with omitted primary antibody, or with depletion of the AQP4 primary antibody by an excess of the specific peptide (Chemicon International, Temecula, CA), did not result in any detectable labelling.

### Immunohistofluorescence: Analysis of AQP4 and ZO-1

Fluorescent microscope images were acquired on a Vslide^®^ slide scanning microscope (MetaSystems, Alltlussheim, Germany). Whole microscope slides were scanned at × 2.5 to adjust focus and to generate a tissue map. Focus and tissue depth were detected based on the DAPI signal. Upper half of brains were scanned using × 10 objective. Images were stitched together to generate large three channel fluorescence images with microscopic resolution. Acquired images were extracted as TIF files using the software Metaviewer® (MetaSystems, Alltlussheim, Germany).

### Immunohistofluorescence: Quantification of AQP4 and ZO-1

ImageJ was used to analyze antibody labelling in the cortical area. Three quadrant ROIs of 550 μm width were manually placed in the medial part of the perilesional area in the cortex, and in the corresponding area on the contralateral side. The thresholds were manually set to each individual antibody, but were then kept fixed throughout the experiment. The staining was automatically quantified with batch processing, using an ImageJ macro (created by us) with thresholding and background subtraction. Three ROIs per section and three sections per animal were used to generate a mean value per animal. All sections for each experiment were stained at the same time, to minimize differences between different staining sessions. Contralateral hemisphere was used for signal normalization, and the results presented as ipsilateral/contralateral values (“ipsi/contra”). In control experiments, multiple localizations were tested to evaluate that the selected ROIs in both hemispheres were representative, and that the areas in the ipsilateral side represented viable tissue. Selected areas were analyzed at a higher magnification (60 × ) to ensure that the tissue morphology appeared intact according to glial fibrillary acidic protein (GFAP)-positive astrocytes, number of nuclei, and the density of blood vessels (data not shown). Any increase of GFAP immunoreactivity within the ROI was taken as an indication that astrocytes in the area were viable. To ascertain that control areas in the contralateral side did not differ from corresponding areas in naive animals, the values of ADC, AQP4, ZO1, GFAP, and IgG were analyzed and found not to differ.

### Statistical analysis

Evaluation of data was performed by an observer blinded for the randomization data. Intraobserver variability was calculated after three measurements, each separated by two weeks for the first five measurements in all settings. No statistical differences between the observations were found, with a variability of 3–7%, depending on the type of measurement. Statistical analysis was performed using a two sided Student's *t* test. All data were normally distributed. *P* values <0.05 were considered statistically significant. Calculations were performed using SPSS 20 (IBM SPSS Statistics, Armonk, NY) and graphs were made using Prism 6.02 (GraphPad Software, La Jolla, CA). Data are expressed as mean ± SD.

## Results

All animals presented normal behavior without apparent neurological deficits after the brain trauma. Physiological parameters (body weight, temperature, saturation, heart rate, and respiratory rate) were within the physiological range throughout the experiment (Table 1).

### Focal brain contusion resulted in brain edema evident in T2 MRI sections

**FIG. 1.**
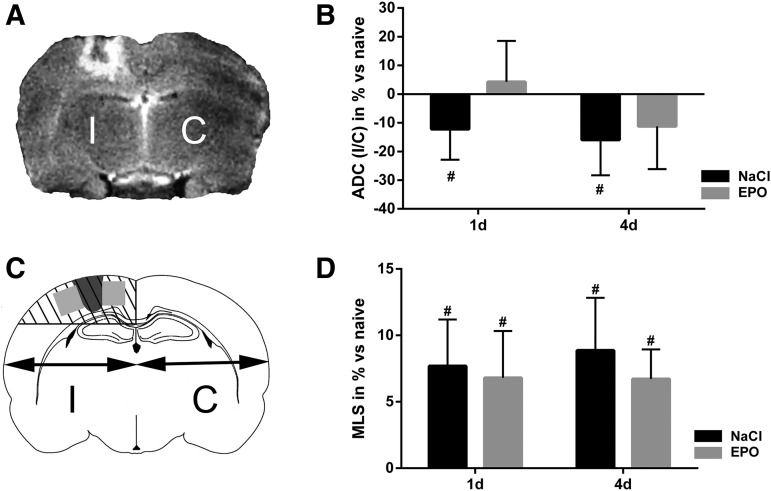
**(A)** Representative T2-weighted coronal MRI section of adult rat brain at day 4 after traumatic brain injury (contusion) in the right hemisphere. The focal injury in the ipsilateral hemisphere (I) is surrounded by a hyperdense area, indicating edema. No hyperdense MRI changes are observed in the contralateral hemisphere (C). **(B)** Relative changes of apparent diffusion coefficient (ADC) in the perilesional region at day 1 and 4 in control animals and erythropoietin (EPO)-treated animals (EPO); ipsilateral/contralateral (I/C) values in percent. ADC I/C was significantly decreased at both day 1 and day 4 after trauma in controls. EPO treatment attenuated the ADC decrease at both time points. Resultant ADC values were similar to ADC in naïve animals without trauma. **(C)** Schematic illustration of a coronal section of a rat brain. Midline shift (MLS) was estimated in percent using the distance between the outer border of the cortex and the middle of the third ventricle in the ipsilateral and contralateral hemispheres, respectively. The dark gray area illustrates the contusion, and the light gray quadrants depict the perilesional regions analyzed in all other experiments. **(D)** MLS at days 1 and 4 after trauma in control animals and EPO-treated animals. A significant MLS toward the uninjured side was present at both time points in both groups; i.e., EPO treatment did not affect MLS. Values presented as mean ± SD. d, days after trauma; #*p* < 0.05)

Evaluation of time course of brain swelling and type of edema development following TBI were assessed by analyzing MRI images with regard to MLS and ADC values. ADC values were measured in the perilesional area lateral to the traumatic brain lesion, and in the corresponding area in the contralateral, uninjured, cortex. Brain MRI images revealed a focal cortical contusion in the right hemisphere of similar magnitude in all animals exposed to TBI. T2-weighted MRI images showed hyperdensity in the perilesional area of the focal injury, indicating the presence of brain edema. No T2 changes were evident in other brain areas in either the ipsi- or contralateral hemispheres (Fig. 1A).

### EPO treatment resulted in less ADC decrease after TBI

ADC values are presented as relative change (in percent) of ipsilateral/contralateral values under the assumption that ADC in naïve, uninjured, animals is equal in both hemispheres.

TBI in the control group resulted in an ADC decrease in the perilesional area by 12% and 15% at days 1 and 4, respectively. EPO treatment prevented the ADC decrease seen in the control group, and did not differ significantly compared with naïve animals at either time point (Fig. 1B). There were no significant changes of ADC in the corresponding areas on the contralateral side at any time.

### EPO did not alter the MLS

The focal TBI caused a significant MLS (Fig. 1C) of the ipsilateral brain hemisphere toward the nontraumatized hemisphere by ∼8% both at day 1 and day 4 after trauma in the control group. MLS was not significantly affected by EPO treatment (Fig. 1D).

### EPO improved BBB morphology

**FIG. 2.**
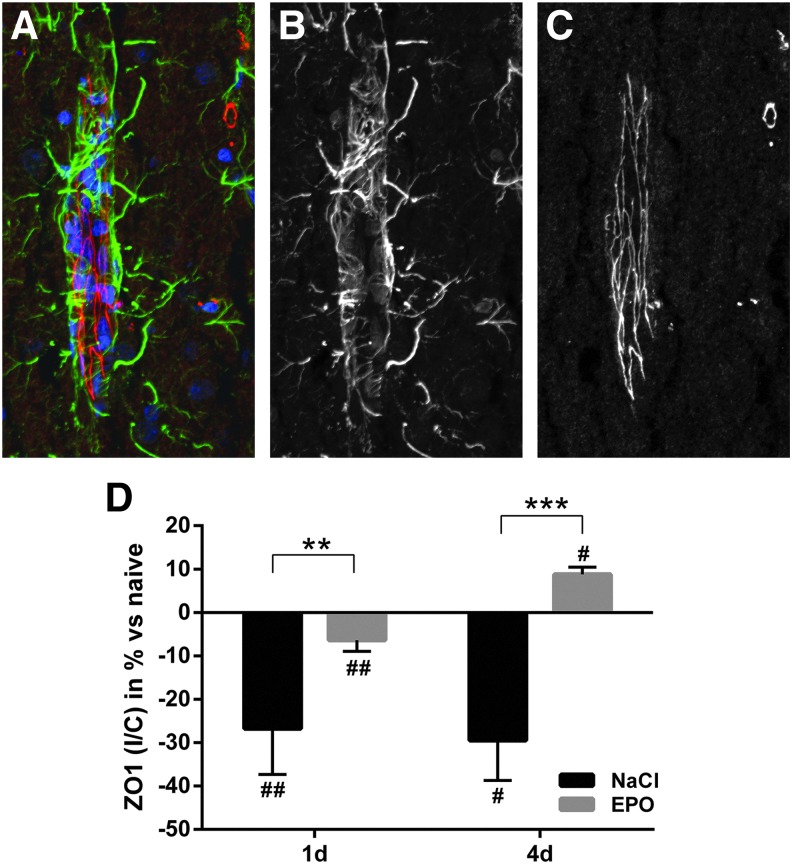
**(A)** High magnification confocal immunofluorescence image of zona occludens 1 (ZO-1) (red), the glial cell marker glial fibrillary acidic protein (GFAP) (green), and the nucleus marker DAPI (blue) from brain area with blood vessels (longitudinal view of blood vessel to the left), to illustrate the specificity and localization of the antibody binding. **(B)** Monochromatic presentation of GFAP immunofluorescence. **(C)** Monochromatic presentation of ZO-1 immunofluorescence. **(D)** Summary data on ZO-1 immunofluorescence in the traumatized, ipsilateral, hemisphere normalized against the contralateral side at days 1 and 4 after trauma in control and erythropoietin (EPO)-treated animals. ZO-1 was significantly reduced after trauma in the control group. EPO prevented the loss of ZO-1 at both time points after trauma. Values presented as mean ± SD. DAPI, 4',6-diamidino-2-phenylindole; #*p* < 0.05, ##*p* < 0.01 compared with naïve animals; **p < 0.01, ***p < 0.001 between groups.

BBB morphology was assessed by analyzing the amount of the endothelial tight junction (TJ) marker ZO-1 in the perilesional area. ZO-1 was decreased by >25% at day 1 and day 4 in control animals. EPO significantly reduced the loss of ZO-1, as animals treated with EPO only had an 8% decrease at day 1 and showed a 10% net increase of ZO-1 at day 4 after trauma (Fig. 2).

### EPO improved BBB integrity

**FIG. 3.**
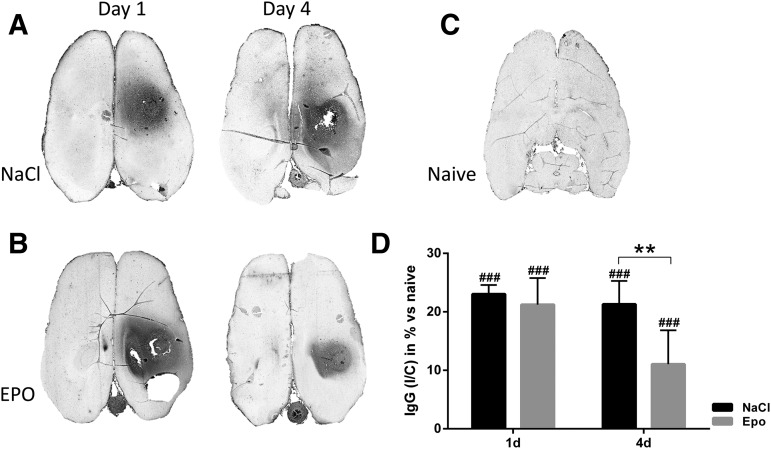
Immunoglobulin (Ig)G immunohistochemistry on representative axial brain sections. **(A)** Control animals day 1 and 4 post-trauma, **(B)** erythropoietin (EPO)-treated animal day 1 and day 4 post-trauma, **(C)** naïve animal without trauma. IgG-positive immunoreactivity (dark area) was observed around the site of the trauma in the right hemisphere. **(D)** Relative changes (in percent) of IgG immunoreactivity at days 1 and 4 after trauma and compared with naïve animals (baseline). The perilesional area is normalized against the contralateral side. IgG was significantly increased in control animals. EPO treatment significantly decreased IgG in the perilesional area at day 4 after trauma. Values presented as mean ± SD. d, days post trauma; I/C, ipsilateral/contralateral; ###*p* < 0.001 compared with naïve animals; **p < 0.01 between groups.

BBB permeability was assessed by quantifying the presence of IgG in the perilesional area. Focal TBI in control animals resulted in an increase of IgG by 23% and 21% at days 1 and 4, respectively. There was no significant change in IgG abundance at day 1 in animals that received EPO; however, EPO treatment significantly decreased IgG in the perilesional area to 11% at day 4 post-injury (Fig. 3).

### EPO attenuated the trauma-induced decrease of AQP4

**FIG. 4.**
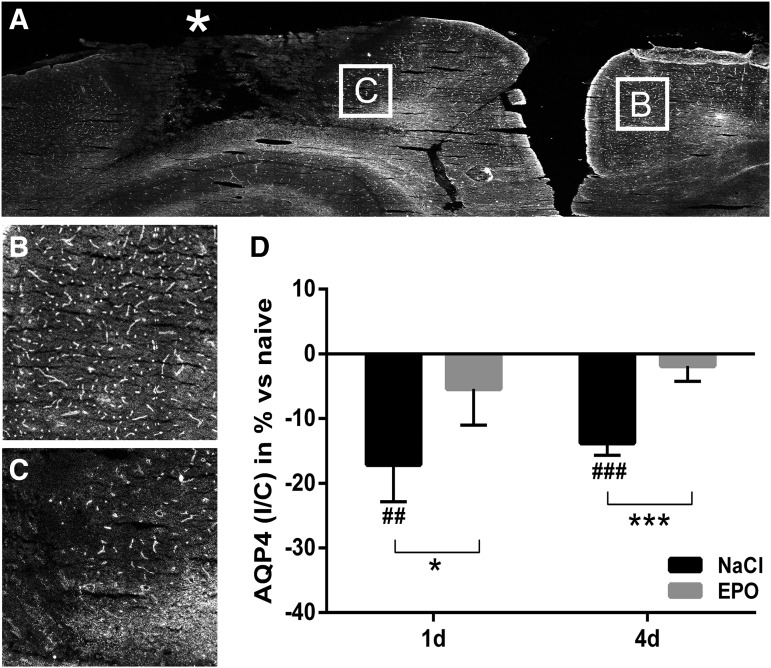
**(A)** Representative monochromatic immunofluorescence image with aquaporin 4 (AQP4) (white) on part of the coronal brain section after blunt controlled parietal trauma (*) in the right hemisphere in a control animal. The core of the injury shows disrupted microscopic structure. Measurements of AQP4 protein abundance were performed in the perilesional area (as shown in Fig. 1C) with a visually intact structure. **(B)** High magnification of representative contralateral and **(C)** perilesional and areas. **(D)** Summary data on relative changes of AQP4 immunofluorescence intensity in the perilesional area at days 1 and 4 after trauma in control and erythropoietin (EPO)-treated animals. AQP4 was significantly reduced at both time points in control animals. EPO treatment normalized AQP4 protein levels in the perilesional area at both days 1 and 4 after trauma. Values presented as mean ± SD. I/C, ipsilateral/contralateral; d, days post trauma; ##*p* < 0.01, ### *p* < 0.001 compared with naïve animals; *p < 0.05, ***p < 0.001 between groups)

In control animals, focal TBI caused a significant decrease of AQP4 protein expression in the perilesional area, as analyzed by fluorescence intensity in ipsilateral/contralateral hemisphere and compared with naïve animals. AQP4 protein was decreased by 17% and 14% at days 1 and day 4, respectively. In EPO-treated animals, the expression of AQP4 did not decrease, and was similar to that in naïve animals (Fig. 4). AQP4 was present in perivascular end-feet surrounding vasculature as confirmed with the endothelial marker rat endothelial cell antigen (RECA)-1 (data not shown). The polarized distribution of AQP4 in predominantly perivascular areas was not overtly changed in either TBI controls or EPO-treated animals as compared with naïve animals.

## Discussion

In this experimental TBI model, we show that daily administration of EPO post injury resulted in improved structure and function of the BBB, preserved AQP4 protein expression in the perilesional area, and MRI changes consistent with decreased cytotoxic brain edema. Mechanisms proposed to be involved in the neuroprotective properties of EPO are mostly attributed to direct effects on neurons,^29–32^ and are expected to be mediated via binding to an alternative EPO-receptor.^33^ This receptor, the innate repair receptor (IRR) is not typically expressed in normal tissue, but is upregulated under hypoxic and ischemic conditions as can be present in brain injury. The IRR activates the Janus-tyrosine-kinase-2 (JAK-2), leading to several nonhemapoetic downstream signaling pathways. Alterations in nitric oxide (NO) production by endothelial nitric oxide synthase (eNOS) and enhanced NO production^34,35^ have also been shown to be protective routes for EPO in animal models such as heart ischemia-reperfusion,^36^ TBI,^37^ and intracerebral hemorrhages.^35^

Several different mechanisms are suggested to be involved in edema development after TBI. According to the traditional view, there is a primary and immediate opening of the BBB after TBI that peaks at 4–6 h and then resolves within 6–7 days after trauma in humans. This opening is considered to be caused by the direct physical trauma, disruption of the BBB,^38,39^ alternations in the expression of AQP4,^40–42^ complement activation,^43^ dysregulation of matrix metalloproteinases,^44^ and release of cytokines.^45^

In a previous study, we showed that the current focal TBI model causes BBB dysfunction as indicated by structural (loss of ZO-1) and functional changes (increased IgG) of the BBB. The findings support a development of vasogenic brain edema as a result of disrupted BBB integrity.^24^

In the present study, we find that administration of EPO after TBI and once daily preserves ZO-1 levels. The effect was observed as early as day 1 after trauma, and persisted at day 4 with a net increase in ZO-1 compared with controls. At day 4, there was a markedly decreased IgG extravasation in the perilesional area in EPO-treated animals. Taken together, the present findings indicate that EPO attenuates trauma-induced BBB dysfunction and thus decreases vasogenic brain edema. ZO-1 is localized at TJ sites and interacts directly with a majority of the transmembrane TJ proteins. In accordance with our findings, dissociation of ZO-1 from the TJ complex has been shown to increase permeability of the BBB.^46–48^ Further, epithelial cells with ZO-1 deficiency do not form TJs because of lack of claudin polymerization,^48^ and establishment of the barrier function is, therefore, delayed.^49^ In TBI, neuroinflammatory mediators such as metalloproteinase-9 cleave extracellular matrix including TJ-associated proteins such as ZO-1. This induces alterations of BBB integrity leading to changes in TJ architecture,^50^ allowing for the development of vasogenic edema.^46^ Our findings thus indicate that treatment with EPO after TBI may preserve BBB function and reduce vasogenic edema by attenuating loss of ZO-1. Such an effect of EPO on BBB disruption in focal TBI has previously not been well characterized. In a weight drop TBI model, EPO was found to decrease permeability to Evans blue, but BBB integrity or morphology was not evaluated.^18^ EPO treatment has, however, been reported to preserve BBB function in other, non-TBI, brain injury models such as cryogenic injury,^51^ MCA occlusion,^52^ and diffuse axonal injury with hypoxia.^53^ The temporal changes in our study show that ZO-1 levels are normalized by day 1 after trauma, whereas the effect on IgG leakage is evident at day 4. We speculate that the focal TBI causes an immediate increase in BBB permeability, and that EPO treatment, because of preserved or early restoration of ZO-1 levels, attenuates a continued IgG leakage as shown by the significantly reduced parenchymal IgG at day 4 after injury. Small amounts of immunoglobulins, including IgG, may be present in normal CNS perivascular tissue, and it has been shown that active transportation of IgG over BBB may occur.^54,55^ However, a significant increase in IgG in brain tissue indicates vasogenic edema as a result of BBB dysfunction.

ADC mapping is recognized to differ between relative changes of the two entities of brain edema; cytotoxic edema, which is associated with low ADC values, and vasogenic edema, which is associated with high ADC values. Here, we found that EPO- treated TBI animals showed ADC values similar to those of naïve animals with no injury. In contrast, control animals subjected to TBI showed a decrease in ADC at both day 1 and 4 post injury, findings consistent with our previous study.^24^ The ADC decrease indicates a presence of cytotoxic edema in the area surrounding the focal injury. EPO treatment, therefore, seems to oppose the development of cytotoxic edema. This effect was observed as early as day 1, and was sustained to day 4 after trauma.

Astrocyte foot processes, with their abundance of AQP4, are susceptible to cytotoxic edema formation. It has been shown that AQP4 is involved in the development of cerebral cytotoxic edema in various pathological conditions. In accordance, AQP4-deficient mice are less prone to develop cytotoxic edema in experimental ischemia and bacterial meningitis.^56,57^ However, the consequences of AQP4 deficiency vary depending on the type of brain edema studied, as AQP4-null mice are more prone to develop vasogenic edema.^58^ In studies of TBI models, there have been reports of both increased^59–61^ and reduced^41,62^ AQP4 protein expression.

In the present model of focal TBI, we found a decrease in AQP4 protein expression in the perilesional area, where the gross and microscopic anatomy otherwise appeared unaffected (present study and an earlier study by Blixt and colleagues^24^). The quantification of AQP4 in the perilesional area was performed using immunohistofluorescence, which allowed us to assess the distribution of AQP4 in the same sections. Interestingly, treatment with EPO counteracted the trauma-induced decrease in AQP4 protein and AQP4 levels were indistinguishable from those in naïve animals without TBI. Moreover, both in control animals and EPO-treated animals, the polarized localization of AQP4 in astrocyte end-feet facing cerebral blood vessels appeared unaffected.

We interpret the absence of ADC decrease after EPO treatment as a decrease in cytotoxic edema. One possible mechanism for the reduced cytotoxic edema may be increased water clearance caused by the preserved protein levels of AQP4 in EPO-treated animals.^63^ Other mechanisms may include downregulation of AQP4 water permeability by EPO^64^ and/or improved cellular ionic and metabolic functions, counteracting water accumulation.^65,66^

We cannot rule out that the attenuation of ADC decrease induced by EPO represents an increase in vasogenic edema; however, we find this unlikely, as the BBB integrity was concomitantly improved by EPO, as shown by a significant decrease in IgG leakage.

Because the craniotomy was not resealed, the current injury model may be considered to resemble a penetrating brain injury leading to a brain contusion. Previous studies^24,26^ have ascertained that this injury model results in edema development and hemisphere enlargement but no brain protrusion, which may be because of the small size of the craniotomy (< 5 mm^2^) as compared with, for example, Dixon's original work (estimated area 80–100 mm^2^).^67^ Pre-hoc, we decided to focus on local effects and evaluation of edema types in the pericontusional area in this study, and decided not to analyze total brain water accumulation or contusion volume. To ensure that the contusions were homogenous and comparable, we compared AQP4, ZO-1, ADC, and IgG values within the same pericontusional areas in the control animals, and found very small deviations.

The focal cortical contusion and edema resulted in a significant MLS of the brain toward the uninjured side. This shift was not significantly affected by EPO treatment. MLS is a crude estimate of brain swelling, and is not considered a sensitive marker of the underlying cause of brain swelling: hyperemia, vasogenic edema, and/or cytotoxic edema. Despite this crudeness, MLS is thought to be a relevant prognostic tool in clinical practice.^68,69^ The lack of statistically significant effect on MLS by EPO treatment here may reflect that this injury estimate has a low sensitivity for changes in small-volume injuries, relatively small sample sizes, or the fact that MLS does not identify shifts of volume between different tissue compartments.

EPO has been shown to be beneficial in multiple experimental models of neurological injury. In a recent study, Operation Brain Trauma Therapy (OBTT) consortium tested EPO in three different TBI models in the rat, including a focal cortical injury model. In the study, a single dose of EPO was administered directly after trauma.^70^ The investigators did not find any effect on either histopathological, cognitive, or biomarker parameters. In the present study of focal TBI, we administered multiple post-injury doses of EPO and observed improved BBB function and an effect on the dynamic edema response following trauma. We did not estimate whether injury volume or neurocognitive function was improved by this dosing regimen of EPO; however, this will be interesting for future studies.

In clinical trials of CNS insults, the results of EPO treatment have been diverse. EPO significantly improved long-term neurological outcome after ischemic stroke in a recent study by Tsai and colleagues,^21^ whereas results have been inconclusive in subarachnoid haemorrhage.^71^ Following moderate or severe TBI, Nichol and coworkers could not show that EPO reduced the number of patients with severe neurological dysfunction on outcome scales 6 months after trauma.^23^ Although not significant, there was an interesting trend toward a favorable outcome with regard to mortality in the EPO treatment group. In a smaller study, using a different EPO dosing regime, Aloizos and colleagues found that EPO decreased mortality and improved neurological outcome at 6 months after severe TBI.^22^

Limitations in the present study include relatively low sample sizes, which may have affected the negative MLS results, and a limited time resolution, which might conceal details in the sequence of events and fluctuations in the estimated parameters in between the analyzed time points. The study does not explore intracellular signaling pathways involved in the effects of EPO on the BBB and brain edema.

## Conclusion

**FIG. 5.**
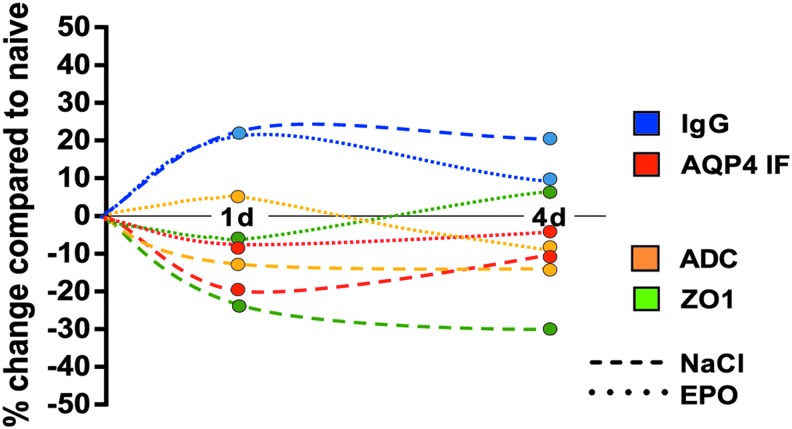
Schematic presentation of relative changes in the perilesional region at days 0, 1 and 4 after trauma in control animals (NaCl) and animals treated with erythropoietin (EPO). Values are compared with those in naïve animals without trauma (point zero) (immunoglobulin [Ig]G, zona occludens 1 [ZO-1], aquaporin 4 [AQP4]), or to the assumption of no difference between the hemispheres (apparent diffusion coefficient [ADC]). The arbitrary continuous timeline is created from the separate measurements for each parameter at the indicated time points. d, days after trauma.

In this experimental TBI model, we show that post-traumatic administration of daily doses of EPO preserved both structural and functional properties of the BBB by preventing loss of the tight junction component ZO-1 and reducing IgG permeability. Further, EPO treatment inhibited the ADC decrease following TBI, indicating that EPO reduced traumatic cytotoxic edema. A summary of the data is presented in Figure 5. In conclusion, the findings in this study support that EPO may have positive effects on traumatic brain edema. Further examination of both dose and timing of EPO administration is warranted.
